# Sequence-dependent effects of ZD1839 (‘Iressa’) in combination with cytotoxic treatment in human head and neck cancer

**DOI:** 10.1038/sj.bjc.6600103

**Published:** 2002-03-04

**Authors:** N Magné, J L Fischel, A Dubreuil, P Formento, S Marcié, J-L Lagrange, G Milano

**Affiliations:** Department of Oncopharmacology, Oncopharmacology Unit, Centre Antoine Lacassagne, 33 Avenue de Valombrose, 06189 Nice Cedex 2, France

**Keywords:** ZD1839, fluorouracil, cisplatin, radiation, head and neck cancer cell lines, combination therapy

## Abstract

Elevated levels of epidermal growth factor receptor in head and neck cancer have been extensively reported, and are correlated with poor prognosis. The combination of cisplatin and 5-fluorouracil is a standard treatment regimen for head and neck cancer, with radiation representing another therapeutic option. Six head and neck cancer cell lines were used to study the cytotoxic effects of combining ZD1839 (‘Iressa’), a new selective epidermal growth factor receptor tyrosine kinase inhibitor, and radiation. Two of the cell lines were also used to study the combination of ZD1839 and cisplatin/5-fluorouracil. Cytotoxic effects were assessed by the MTT test. The results indicated that ZD1839 applied before radiation gave the best effects (*P*=0.002); an effect that was strongest in those p53-mutated cell lines that express the highest epidermal growth factor receptor levels. The effects of ZD1839 with cisplatin and/or 5-fluorouracil were sequence dependent (*P*<0.003), with the best results achieved when ZD1839 was applied first. For the triple combinations, ZD1839 applied before cisplatin and 5-fluorouracil resulted in a slight synergistic effect (*P*=0.03), although the effect was greater when ZD1839 was applied both before and during cytotoxic drug exposure. In conclusion, ZD1839 applied before radiation and before and/or during cisplatin/5-fluorouracil may improve the efficacy of treatment for head and neck cancer.

*British Journal of Cancer* (2002) **86**, 819–827. DOI: 10.1038/sj/bjc/6600103 www.bjcancer.com

© 2002 Cancer Research UK

## 

Epidermal growth factor receptor (EGFR) has been particularly well studied in head and neck cancer (HNC) because of its association with the cellular mechanisms involved in tumour progression. High levels of EGFR expression have been correlated with poor prognosis (Santini *et al*, 1991; Dassonville *et al*, 1993). EGFR signalling has been found to control not only cell growth, but also angiogenesis and DNA repair (Wheeler *et al*, 1999; Schlessinger, 2000), and has recently been assessed as an innovative target in cancer therapy and particularly in HNC. To date, clinical studies in this area have involved the administration of cetuximab (C225, a chimeric monoclonal antibody), either alone or in combination with chemotherapy or radiotherapy, to patients with advanced head and neck squamous cell carcinoma (Ezekiel *et al*, 1999; Huang *et al*, 1999; Mendelsohn *et al*, 1999; Baselga *et al*, 2000; Huang and Harari, 2000; Milas *et al*, 2000). More recently, a wide range of small-molecule tyrosine kinase inhibitors have been developed, and one of the most advanced in this class of compounds is ZD1839 (‘Iressa’), which is a quinazoline derivative. This agent interacts specifically with the highly conserved ATP binding site of the tyrosine kinase domain of EGFR, resulting in inhibition of ligand-induced EGFR activation, and blockade of signal transduction pathways (Ciardiello *et al*, 2000; Sirotnak *et al*, 2000).

The cisplatin/5-fluorouracil (5-FU) regimen is considered to be a standard chemotherapy regimen in the treatment of advanced HNC as part of an organ-conserving strategy (Taylor *et al*, 1997; Brizel *et al*, 1998; Calais *et al*, 1999). Another standard treatment is the combination of external beam radiation therapy with surgical extirpation (Bensadoun *et al*, 1998; Fu *et al*, 2000; Vokes *et al*, 2000). Despite the effectiveness of these treatments, the survival rates vary according to the tumour site and stage, and globally, prognosis remains poor (Vokes *et al*, 1993). Thus, one interesting and promising research direction for altering the natural history of HNC could be a molecular-targeted therapy against EGFR in association with one of the standard therapeutic strategies. Experimental data have indicated that application of EGFR-targeting agents can not only slow down cell proliferation, but also improve apoptotic capacities and decrease DNA repair. These observations suggest that EGFR targeting can lead to chemo- and radiosensitisation and recent experimental results tend to confirm this view (Huang *et al*, 1999).

The aim of this study was two-fold: firstly, to explore the sequence-dependent cytotoxic effects of combining ZD1839 with radiation using a panel of six human head and neck squamous cell carcinoma cell lines; and secondly, to investigate the sequence-dependent cytotoxic effects of combining ZD1839 with cisplatin and/or 5-FU on two of the cell lines from the panel. This preclinical work was undertaken to serve as a rationale to support ongoing clinical investigations of ZD1839 in HNC patients.

## MATERIALS AND METHODS

### Chemicals

ZD1839 was kindly provided by AstraZeneca. A 50 mM working solution in dimethysulphoxide (DMSO) was prepared before use. Human recombinant ^125^I-EGF (ref IM 196, specific activity 4514×10^10^ Bq mmol^−1^, 92.5×10^4^ Bq per 250 μl) and unlabelled human recombinant EGF (ref ARN 5100) were obtained from Amersham. Dulbecco's modification of Eagle's medium (DMEM), RPMI 1640 and glutamine were purchased from Whittaker (Verviers, Belgium). Foetal bovine serum (FBS) was obtained from Dutscher (Brumath, France). Penicillin and streptomycin were from Meyrieux (Lyons, France). Transferrin and insulin were purchased from Flow (Irvine, Scotland). Bovine serum albumin (BSA), 3-(4-5-dimethylthiazol-2-yl)-2,5-diphenyl tetrazolium bromide (MTT) and DMSO were purchased from Sigma (St Quentin Fallavier, France).

### Cell lines

Six HNC cell lines of human origin were used (CAL27, CAL33, CAL60, CAL166, Hep-2, Detroit562) (Table 1

**Table 1:**
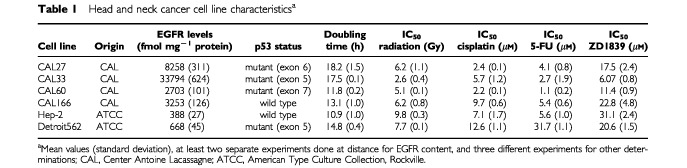
Head and neck cancer cell line characteristics^a^

). Cells were routinely cultured in DMEM supplemented with 10% FBS, 2 mM glutamine, 600 μg l^−1^ insulin, 500 μg l^−1^ transferrin, 50 000 units l^−1^ penicillin and 80 μM streptomycin in a fully humidified incubator (Sanyo, Japan) at 37°C in an atmosphere containing 8% CO_2_.

### EGFR assay

EGFR expression was assayed by competitive analysis and Scatchard plots as previously described in human cancer cell lines (Olivier *et al*, 1990). Cells were grown in 24-well plates (10^5^ cells per well) in 10% FBS-DMEM at 37°C. At 80–90% confluence, cells were rinsed three times with 500 μl RPMI 1640 containing 0.1% BSA at 2–4°C (plates placed on a tray with ice). Plates were then incubated for 30 min with the same medium (500 μl per well) at 4°C. Cells were first screened for their capacity to bind EGF specifically. Total binding was measured after incubation with 0.2 nM ^125^I-EGF (3 h, 4°C, 0.1% BSA–RPMI); non-specific binding was measured in the presence of an excess of unlabelled EGF (20 nM). With no exception, the six tested cell lines exhibited EGF binding and their precise EGFR content was then measured. Cells were incubated in the RPMI medium for 3 h, at 4°C in the presence of various concentrations of ^125^I-EGF (0.01, 0.02, 0.04, 0.08, 0.12, 0.18, 0.20 nM); for higher EGF concentrations, cells were incubated with 0.2 nM ^125^I-EGF with increasing concentrations of unlabelled EGF (0.05, 0.1, 0.2, 0.4, 0.8, 1.6, 3.2, 6.4, 20, 200 nM). Plates were placed on ice to stop the reaction, and the supernatant was removed from each well. Cells were washed twice with phosphate-buffered saline (PBS) containing 0.1% BSA (4°C, 500 μl per well). After removal of the supernatant, cells were solubilised in 1 M NaOH at 37°C (500 μl per well for 30 min). The radioactivity of each well was determined by gamma counting. Results were expressed in fmol per mg cell protein. Scatchard analysis was used to calculate the number of receptor sites per cell (N) and the dissociation constant (K_d_). Each point of every Scatchard plot was performed in quadruplicate. Cells were counted in four wells run in parallel, resuspended in 200 μl PBS at room temperature and counted with a haemocytometer. Experiments were performed in duplicate only, because of the intrinsic reproducibility of the assay (coefficient of variance=7.3%, *n*=4).

### Determination of p53 status

DNA was extracted from all head and neck cell lines of the panel. Exons 4 to 8 were screened for mutations by use of denaturing gradient gel electrophoresis in accordance with the method described by Hamelin *et al* (1993) for exons 5, 7, and 8, and the method of Gulberg *et al* (1997) for exons 4 and 6. Exon 9 was screened for mutations by the method described by Cabelguenne *et al* (2000). Polymerase chain reaction (PCR) amplification products were loaded onto a 6.5% polyacrylamide gel that contained an appropriate gradient of urea and formamide. After electrophoresis, gels were stained with ethidium bromide. Tumours that showed an electrophoresis variant pattern were amplified and sequenced for each variant exon. PCR products were purified with QIAquick PCR Purification Kit (QIAGEN SA, Courtabeuf, France) and sequenced on both strands on an ABI 310 genetic analyser (PE Applied Biosystems, Courtabeuf, France). A Big Dye Terminator sequencing kit (PE Applied Biosystems) was used in accordance to the manufacturer's instructions; this step was followed by ethanol precipitation to remove non-incorporated dyes. Sequences were analysed by Sequence Analysis 3.0 (PE Applied Biosystems).

### Evaluation of radiotoxicity

Cells were irradiated with gamma rays during exponential cell growth as monolayers in 96-well microtitration plates using a ^60^Co unit at a dose rate of 1 Gy min^−1^. Dose-effect curves were established for all six head and neck cell lines using a total of 10 doses (0.5, 1, 2, 3, 4, 6, 8, 10, 12, 15 Gy). Sequence-dependent cytotoxic effects of binary combinations of ZD1839 (0.2–200 μM) with ionising radiation were then evaluated using eight doses of ionising radiation, ranging from 0.5 to 10 Gy for CAL27 and CAL33, and from 2 to 15 Gy for CAL60, CAL166, Hep-2 and Detroit562 (the radiation doses were selected from the above experiments to encompass more precisely the zone of sensitivity). Cells were maintained in DMEM supplemented with 10% FBS during all radiation exposures, which were performed at room temperature.

### Determination of the cytotoxic effects of radiation alone

Cells were seeded in 96-well microtitration plates (100 μl per well) to obtain exponential growth for the whole duration of the experiment: initial cell densities were 2500 (CAL27 and Hep-2), 3000 (CAL33 and Detroit562), 4500 (CAL166) and 5000 (CAL60) cells per well.

The cells were exposed to ionising radiation 48 h later (dose previously described) and the MTT assay was performed 48 h after that. Control cells received no radiation treatment.

#### Determination of cytotoxicity using ZD1839 in combination with radiation

Cells were seeded in 96-well microtitration plates (100 μl per well) to obtain exponential growth for the whole duration of the experiment (initial cell density is given above). In each plate, one ZD1839 concentration and one radiation dose were tested. Only half of each plate was irradiated in order to measure on the same plate the effects of ZD1839 alone (20 wells), radiation alone (five wells), ZD1839 combined with radiation (20 wells) and a control (no radiation, no ZD1839; five wells).

Forty-eight hours after seeding the cells, three different sequences of radiation and 48-h incubations of ZD1839 were compared: (a) ZD1839 (48 h) followed by radiation, then medium for 48 h; (b) concomitant ZD1839 (48 h) and ionising radiation (radiation delivered in the middle of ZD1839 exposure), followed by medium for 48 h; (c) ionising radiation prior to ZD1839 (48 h), then medium for 48 h.

Growth inhibition was assessed 168 h after cell seeding by using the MTT test as described below (Carmichael *et al*, 1987). Cells were washed with PBS and incubated with MTT; after 2 h of exposure, MTT was released and fixation was revealed by the addition of DMSO (100 μl). Absorbance at 450 nm was measured using a microplate reader (Labsystems, Helsinki, Finland). Results were expressed as the relative percentage of absorbance compared with controls without drug. Cell sensitivity to the tested drugs was expressed by IC_50_ (concentration leading to 50% cell survival). Triplicate determinations were made in separate experiments.

### Evaluation of cisplatin and/or 5-FU cytotoxicity

Cells were seeded in 96-well microtitration plates (100 μl per well) to obtain exponential growth for the duration of the experiments (initial cell density was 2500 and 3000 cells per well for Hep-2 and CAL33, respectively). Forty-eight hours after seeding, cells were exposed to ZD1839 and cisplatin and/or 5-FU in a variety of sequences. In all cases, cells were exposed to ZD1839 for 48 h, and the cisplatin/5-FU sequence was constant (cisplatin applied for 2 h prior to exposure to 5-FU for 48 h). The sequence of ZD1839 varied: (a) ZD1839 (48 h) followed by cisplatin and/or 5-FU; (b) concomitant ZD1839 and cisplatin and/or 5-FU (the exposure to ZD1839 was 50 h); (c) ZD1839 prior to and during cisplatin/5-FU exposure (ZD1839 was given for 48 h before cisplatin/5-FU and then for 50 h concomitant with cisplatin/5-FU, making 98 h in total) followed by medium for 48 h; (d) cisplatin/5-FU prior to ZD1839 followed by medium for 48 h. Eleven concentrations were tested for each drug: ZD1839 0.2–200 μM; cisplatin 0.1–200 μM; 5-FU 0.22–220 μM. The cisplatin/5-FU combination was tested at a constant concentration ratio of the drugs for a given cell line, the ratio being dictated by the drug sensitivity and close to the ratio of the IC_50_ for each drug.

Thereafter, growth inhibition was assessed 48 h after the end of the experiment (in medium alone) by the MTT test described above. Experimental conditions were tested in sextuplicate (six wells of the 96-well plate per experimental condition) and separate experiments were performed in triplicate. The dose-effect curves were analysed using Prism software (GraphPad Software, San Diego, CA, USA).

### Combination index (CI) calculations and determination of the potentiation factor

The cytotoxic effects obtained with the different ZD1839/cisplatin/5-FU combinations were analysed according to the Chou and Talalay (1984) method on Calcusyn software (Biosoft, Cambridge, UK). Interaction between the double combinations (ZD1839 plus either cisplatin or 5-FU), or the triple combinations (ZD1839 plus cisplatin and 5-FU) was assessed by means of an automatically computed combination index (CI). CI was determined at 50 and 75% cell death, and was defined as follows:


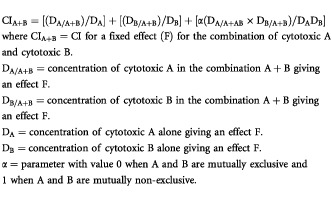


The combination index indicated: synergism <0.8; additivity >0.8 and <1.2; antagonism >1.2; slight synergistic and additive cytotoxic activity for values of 0.8 and 1.2, respectively.

The cytotoxic effects obtained with the different ionising radiation/ZD1839 combinations were also analysed according to the Chou and Talalay (1984) method as described above. CIs were determined at 50 and 75% cell death. The application of the Chou and Talalay model recommends the use of cytotoxic agents at a fixed dose ratio (for example, the ratio of their IC_50_s). However, in the case of ZD1839 and radiation, this strategy would not have been feasible because the dose-effect curves of each cytotoxic agent acting alone are too different (e.g. IC_90_/IC_10_=50 for ZD1839 and 2.8 for radiation (CAL33 cell line)). Consequently, ZD1839 and radiation were combined at equitoxic effects (i.e. doses were applied in the combination that would have produced the same cytotoxic effect if given alone); it follows that the ratio between the concentration of ZD1839 and the radiation dose varied from 4×10^−7^ up to 2×10^−5^ when progressing from low to high cytotoxic effect. When using non-constant ratios between the doses, the Chou and Talalay Calcusyn programme does not allow direct calculation of the CIs at a fixed final cell death (50 and 75% in the present case). Thus, in order to compare CI at fixed values, i.e. CI_50_ or CI_75_, we graphically interpolated between values obtained from the experimental points (11 points generated by the programme between 10 and 90% of final death).

The potentiation factor of ionising radiation by ZD1839 was defined as the ratio of the IC_50_ of ionising radiation alone to the IC_50_ of the combination (ZD1839 plus ionising radiation); higher potentiation factors indicate greater cytotoxicity. Potentiation factor I was obtained using sequence I (ZD1839 (48 h) followed by radiation, then medium for 48 h). Potentiation factor II used sequence II (concomitant ZD1839 (48 h) and ionising radiation, radiation delivered in the middle of the ZD1839 exposure), and potentiation factor III used sequence III (ionising radiation prior to ZD1839 (48 h), then medium for 48 h).

### Statistical analysis

For the panel of cell lines, the relationship between EGFR expression and response to ionizing radiation was analysed by plotting IC_50_ values for radiation against the respective EGFR content. The Friedman non-parametric rank test was used to analyse the impact of the sequence ZD1839/cisplatin/5-FU (CI values) or ZD1839/radiation and to compare the different sequences combining ZD1839 and radiation on the basis of the potentiation factors. The correlation coefficients (*r*) and the *P*-values were computed using SPSS software (Chicago, IL, USA). A *P*-value of less than 0.05 was considered to be statistically significant.

## RESULTS

The study panel of human squamous cell carcinoma cells showed substantial variability in EGFR expression, ranging from 388 (Hep-2) to 33 794 (CAL33) fmol per mg cell protein. Four of the six cell lines were found to be mutated for p53 gene; CAL166 and Hep-2 were p53 wild type (Table 1).

**Figure 1:**
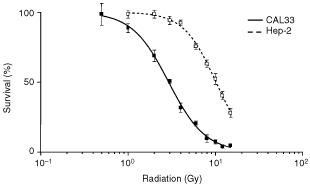
Dose-survival curves following ionising radiation for Hep-2 and CAL33 cells.

**Figure 2:**
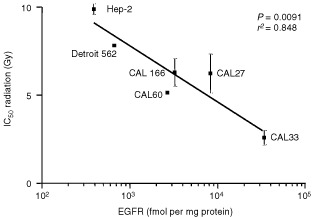
Correlation between cell sensitivity to ionising radiation and EGFR content.

Hep-2 cells were the most radioresistant (IC_50_ 9.8±0.3 Gy), and CAL33 cells the most radiosensitive (IC_50_ 2.6±0.4 Gy). Figure 1

shows dose-survival curves following irradiation for Hep-2 and CAL33 cells. Over the panel of cell lines, growth inhibition was more pronounced in EGFR-overexpressing cell lines than it was in cell lines expressing low levels of EGFR (Table 1). A statistically significant linear inverse correlation was found between EGFR cellular content and IC_50_ for radiation (*P*=0.0091, *r*^2^=0.848) (Figure 2

). No statistically significant correlation was found between EGFR levels and cell doubling time for the six head and neck cell lines (*P*=0.093, *r*^2^=0.548).

**Figure 3:**
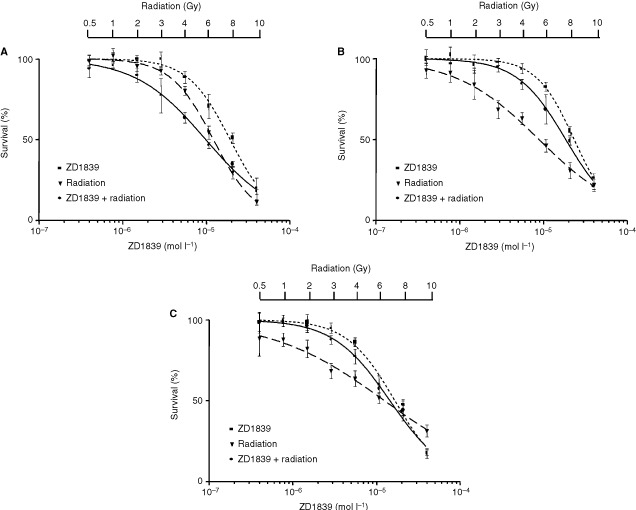
Dose-effect curves for the different ZD1839/radiation exposure combinations for CAL33 cells: (**A**) sequence I, (**B**) sequence II, (**C**) sequence III. For each combination sequence of ZD1839 and radiation, the dose-effect curve of the combination was compared with the dose-effect curves of ZD1839 alone and radiation alone. Radiation dose ranged from 0.5 to 10 Gy.

Typical dose-effect curves for the different ZD1839/radiation exposure combinations are shown in Figure 3

for CAL33. In all cell lines, pretreatment with ZD1839 followed by ionising radiation (sequence I) produced a leftward shift of the concentration-response curves, indicating a greater cytotoxic effect. The CI values computed at 50 and 75% cell lethality are given in Table 2

**Table 2:**
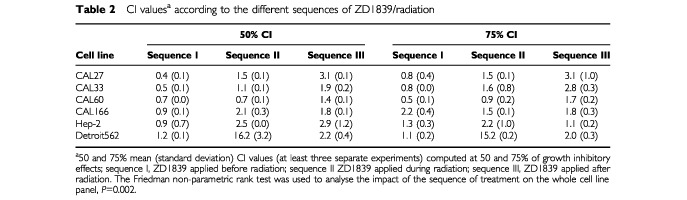
CI values^a^ according to the different sequences of ZD1839/radiation

**Figure 4:**
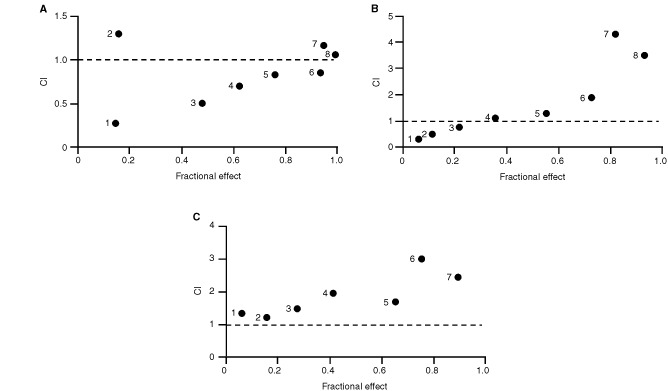
Typical examples of CI/fractional effects curves for CAL33 cells: (**a**) sequence I, (**b**) sequence II, (**c**) sequence III.

. It appears that ZD1839 applied 48 h before radiation (sequence I) led to lower CI values in all head and neck cell lines (*P=*0.002). More precisely the best synergistic effects were observed in the p53-mutant cell lines that express the highest EGFR levels (CAL27, CAL33, CAL60). Typical examples of CI/fractional effect curves are illustrated in Figure 4

for CAL33.

Table 3

**Table 3:**
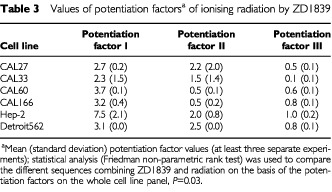
Values of potentiation factors^a^ of ionising radiation by ZD1839

gives the potentiation factors for ZD1839 and ionizing radiation. Statistical analyses indicated that the relative contributions of the two treatments are significantly different depending upon the sequence (*P*=0.001). The greatest contribution to the overall cytotoxicity of the combination came from ZD1839 applied 48 h before irradiation (sequence I; highest relative contribution) and the smallest from ZD1839 following irradiation (sequence III).

The order of association between ZD1839 and combined cytotoxic drugs had a significant impact on their interaction. For both cell lines, application of the Wilcoxon test shows that the association between ZD1839 and cisplatin alone gives supra- additive effects more frequently when ZD1839 is applied first; the same conclusions are true for ZD1839 combined with 5-FU, with mainly synergistic effects when ZD1839 is given before 5-FU (*P* <0.003) (Table 4

**Table 4:**
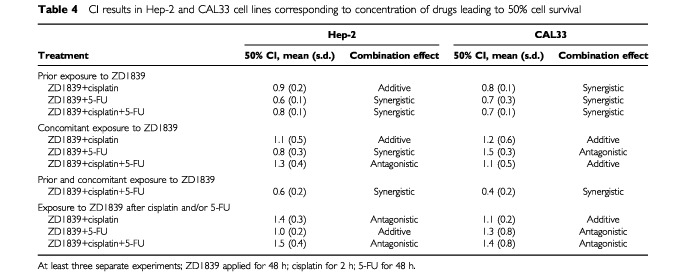
CI results in Hep-2 and CAL33 cell lines corresponding to concentration of drugs leading to 50% cell survival

). When ZD1839 was applied during or after each of these drugs, effects were usually additive or antagonistic. With the triple combination (ZD1839, cisplatin, 5-FU), the effects were synergistic when ZD1839 was applied first, in both Hep-2 and CAL33 cells (*P*=0.003) (Table 4). In contrast, when ZD1839 was applied during or after cisplatin/5-FU there were antagonistic or additive effects. Interestingly, the optimal result (lowest CI value) was obtained with sequence III, in which ZD1839 is applied before and during cisplatin/5-FU (Table 4). Typical dose-effect curves comparing sequences I and III are given in Figure 5

**Figure 5:**
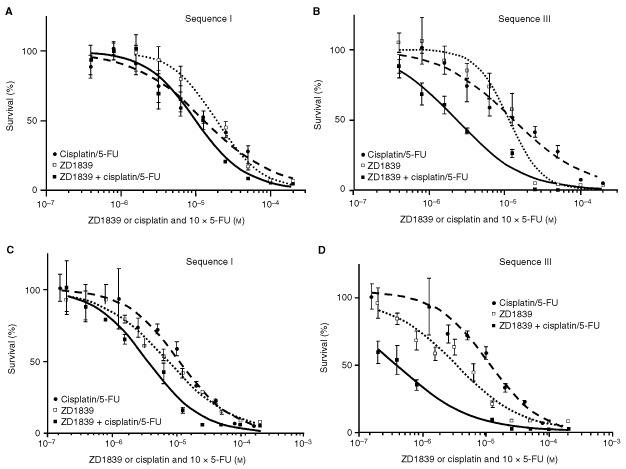
Typical dose-effect curves comparing sequences I and III for (**A**, **B**) Hep-2 and (**C**, **D**) CAL33. Sequence I consisted of ZD1839, followed by cisplatin/5-FU for 48 h; sequence III was ZD1839 applied first and also during the cisplatin/5-FU exposure, followed by medium for 48 h.

for Hep-2 and CAL33.

## DISCUSSION

Squamous cell carcinomas in the aerodigestive tract remain a significant public health problem (Vokes *et al*, 1993). Even with multimodal therapy, the long-term remission rate for these cancers is only around 50–60%. External beam radiotherapy represents one of the main therapeutic tools in HNC, producing major antitumour responses when used in conjunction with both cisplatin and 5-FU in either induction or adjuvant treatment settings (Vokes *et al*, 2000). A recent meta-analysis has shown that a substantial survival benefit can be obtained by chemo-/radiotherapy combination in advanced HNC (Pignon *et al*, 2000). The challenge in this pathology is to incorporate new agents into established regimens in order to obtain a significant impact on overall outcome. Over the past decade, comprehensive data have been accumulated that strongly support a role for EGFR and its ligands in tumour development and growth. This is particularly true for squamous cell carcinoma of the head and neck, where EGFR is considered to be one of the major prognostic factors (Santini *et al*, 1991; Dassonville *et al*, 1993; Salomon *et al*, 1995; Grandis *et al*, 1996, 1998; Maurizi *et al*, 1996). As a result, EGFR has become an attractive target for novel anticancer therapies, particularly in HNC.

Previous studies have shown that stimulation of the EGFR signalling pathway leads to radioresistance both *in vitro* and *in vivo* (Wollman *et al*, 1994; Miyaguchi *et al*, 1998; Akimoto *et al*, 1999; Dent *et al*, 1999). Interestingly, in the present study, we observed a strong positive association between EGFR level and radiosensitivity, which is in agreement with previous results of Bonner *et al* (1994, 1998, 2000). Among a panel of human tumour cell lines, these authors found that the cell line expressing the lowest EGFR levels demonstrated the greatest radioresistance, and that the greatest growth inhibition was observed for the cell line with the highest EGFR content. In addition, there are some *in vitro* reports suggesting an EGF-related radiosensitisation. For instance, Kwok and Sutherland (1989, 1991) have shown that EGF-related radiosensitization may be EGFR-density dependent. A link between the presence of transforming growth factor alpha (TGFα) and radiosensitivity was also suggested by the clinical data reported by Sauter *et al* (1994) and Wen *et al* (1996). More precisely, Sauter *et al* (1994) found that low plasma TGFα levels were associated with radioresistance in patients with squamous carcinoma of the tongue. Wen *et al* (1996) reported that the recurrence rates of early laryngeal cancer treated with radiotherapy correlated with TGFα but not with EGFR. Thus, it appears that not only EGFR but also the tumoural content of its specific ligands may play an important role in radiosensitivity. Cell doubling time and EGFR content were not found to be correlated in the present study. Thus the impact of EGFR on radiosensitivity is not explicable by EGFR-dependent modifications of cell kinetic characteristics, which could enhance the action of irradiation. The classical view of the cellular effects of ionising radiation consists of an initial event involving the induction of DNA damage, in addition to the activation of several intracellular signalling cascades that have commonly been regarded as mitogenic, including the Raf-MEK-Erk kinase cascade (Todd *et al*, 1999). DNA damage is modulated by metabolic DNA repair processes. Interestingly, Bandyopadhyay *et al* (1998) have shown that EGFR-mediated signalling may control the activity of DNA-PK, an enzyme directly involved in DNA damage repair; more precisely, from their work, high EGFR expression could maintain a high level of signalling, which would be associated with high basal levels of DNA repair capacity. However, this does not appear to explain the observations reported here. An approach used by Lewis *et al* (2000), examining the whole pattern of MAP kinase pathway signalling targets, could help to elucidate the molecular origins of the present observation of a radiosensitivity linked to high tumoural EGFR levels.

A strong positive interaction between irradiation and ZD1839, when the ZD1839 was applied before the radiation, was demonstrated in the present study (Tables 2 and 3). This could be explained by an intrinsic activity of ZD1839 as radiosensitiser, as has previously been shown for cetuximab, another EGFR-targeting drug (Ezekiel *et al*, 1999; Huang *et al*, 1999; Mendelsohn *et al*, 1999; Baselga *et al*, 2000; Huang and Harari, 2000; Milas *et al*, 2000), and also for trastuzumab (Herceptin™), a HER-2-targeting drug (Pegram *et al*, 1998; Pietras *et al*, 1999; Pegram and Slamon, 2000). Trastuzumab increased the radiosensitivity of HER-2-overexpressing MCF7 breast cancer cells as measured by *in vitro* colony-forming assays, and the combination of trastuzumab and radiation showed synergistic tumour reduction in nude mice (Pietras *et al*, 1999). According to Pietras *et al* (1999) the mechanism of radiosensitization appears to involve both cell cycle regulation and DNA repair. Interestingly, the application of ZD1839 in our study was found to increase the percentage of cells in the G1 phase (results not shown), which are particularly sensitive to radiation (Teyssier *et al*, 1999). Thus, there are several potential mechanisms of radiosensitisation by inhibitors of the EGFR family; they may include favourable cell cycle reorganisation and/or abrogation or attenuation of signals required for survival during cell cycle arrest. Marked antagonistic effects were observed when exposing cells to ZD1839 after irradiation (sequence III). This observation may be related to the fact that irradiation is able to upregulate EGFR phosphorylation in a similar manner to EGF (Wan *et al*, 2001) and thus create the circonstances of opposite effects to ZD1839.

Two cell lines of the initial panel of six were considered for analysis of the association between ZD1839 and cytotoxic drugs. We have recently demonstrated an inverse correlation between p53 content (representative of p53 mutations) and EGFR levels in a group of HNC patients (Etienne *et al*, 1999). Thus, the two cell lines were selected because they are representative of human HNC characteristics on the basis of their p53 status and EGFR status: CAL33 is p53 mutant and has high EGFR expression, and Hep-2 is p53 wild type and has low EGFR expression. We observed synergistic effects when ZD1839 was applied before cisplatin and/or 5-FU in both cell lines (Table 4). The impact of ZD1839 on the effects of the combined drugs was sequence dependent. The highest synergistic effects were obtained when ZD1839 was applied before and during exposure to cytotoxic drugs (Table 4). Previous *in vivo* and *in vitro* studies have shown that the anti-EGFR receptor monoclonal antibody C225 can potentiate the effects of a number of chemotherapeutic agents, including doxorubicin, paclitaxel and cisplatin (Ezekiel *et al*, 1999; Mendelsohn *et al*, 1999; Huang *et al*, 1999; Baselga *et al*, 2000; Huang and Harari, 2000; Milas *et al*, 2000). The mechanisms by which receptor blockade by C225 augments the cytotoxic effect of anti-neoplastic agents was attributed by these authors to cell cycle effects and an enhanced capacity for apoptosis by C225. The antiproliferative activity of ZD1839 combined with a number of cytotoxic drugs (cisplatin, carboplatin, oxaliplatin, paclitaxel, docetaxel, doxorubibin, etoposide, topotecan and raltitrexed) has been recently assessed in a variety of human cancer cell lines (Ciardiello *et al*, 2000; Sirotnak *et al*, 2000). The co-administration of ZD1839 was found to enhance the growth-inhibitory effects of all cytotoxic drugs tested. The present data confirm this interesting property of ZD1839 as a strong radio- and chemosensitizer. In addition, the present study brings particular focus to the importance of the combination sequence: it is shown that synergy is not a rule and that some antagonistic effects may result from inappropriate sequences, e.g. where ZD1839 is applied after the cytotoxic drugs (Table 4). In conclusion, the present investigations into the combinations of ZD1839 and radiation, and ZD1839 and cisplatin/5-FU, led to similar findings of sequence-dependent synergy. Although an unavoidable gap does exist between the bench and the bed side, these data may be useful for the design of future clinical trials combining ZD1839 and cytotoxic agents.
